# Mycelium-Based Composite Materials: Study of Acceptance

**DOI:** 10.3390/ma16062164

**Published:** 2023-03-08

**Authors:** Agata Bonenberg, Maciej Sydor, Grzegorz Cofta, Beata Doczekalska, Klaudia Grygorowicz-Kosakowska

**Affiliations:** 1 Institute of Interior Design and Industrial Design, Faculty of Architecture, Poznan University of Technology, 61-131 Poznań, Poland; 2 Department of Woodworking and Fundamentals of Machine Design, Faculty of Forestry and Wood Technology, Poznań University of Life Sciences, 60-637 Poznań, Poland; 3 Department of Chemical Wood Technology, Faculty of Forestry and Wood Technology, Poznań University of Life Sciences, 60-637 Poznań, Poland

**Keywords:** mycelium-based composites, mycomaterials, bio-design, interior design, furniture design, aesthetics, eco-aesthetics, fast fashion, customer perspective

## Abstract

Mycelium-based composites (MBCs) are alternative biopolymers for designing sustainable furniture and other interior elements. These innovative biocomposites have many ecological advantages but present a new challenge in aesthetics and human product acceptance. Grown products, made using living mycelium and lignocellulosic substrates, are porous, have irregular surfaces and have irregular coloring. The natural origin of these types of materials and the fear of fungus can be a challenge. This research investigated the level of human acceptance of the new material. Respondents were students of architecture who can be considered as people involved in interior design and competent in the design field. Research has been performed on the authors’ prototype products made from MBCs. Three complementary consumer tests were performed. The obtained results measured the human reactions and demonstrated to which extents products made of MBCs were “likeable” and their nonobvious aesthetics were acceptable to the public. The results showed that MBC materials generally had a positive or not-negative assessment. The responses after the pairwise comparison of the MBC with wall cladding samples pointed out the advantage of ceramic reference material above the MBC based on an overall assessment. The respondents also believed that the chamotte clay cladding would be easier to fit into the aesthetics of a modern interior and would in better accordance with its style. Although the MBC was less visually appealing, the respondents nevertheless found it more interesting, original, and environmentally friendly. The experiments suggested that the respondents had double standards regarding MBCs. MBCs were generally accepted as ecological, but not in their own homes. All of these results support current and future applications of MBCs for manufacturing items where enhanced aesthetics are required.

## 1. Introduction

Chitin has a number of desirable properties, including being biodegradable and biocompatible, which makes it an attractive alternative to synthetic polymers. In its raw form, chitin is brittle and difficult to process. However, chitin can be processed into different forms, including chitosan [1] and chitin nanofibrils [2]. Chitin nanofibrils of fungi can be used as an reinforcement in biocomposites for furniture and building materials [3]. The priority date of first patent on mycelium-based composites (MBCs) dates back to 2007 [4], while the scientific publications started in 2012 [5]. Since 2013, more than 30 review articles have been published describing various aspects of this type of material, including production [6], applications and properties [7], electronic applications [8], architecture applications [9], patents related to MBCs [10], furniture and art applications [11], a sustainable development [12], and proper selection of material-generating species of fungi [13].

Following the idea of material-generating use of fungi, the authors have begun researching MBCs in interior design use. The analysis indicates that MBCs could become an alternative to sustainable furniture and other interior design materials, despite some known engineering flaws, such as the low ability to transfer tensile forces and high hygroscopicity, resulting in low outdoor durability [11,13]. The use of fungi in the production of MBC usually raises concerns about the health impact, but when compared to MDF, in which formaldehyde or other chemicals are used, MBCs seem to be a safer option [14].

In addition to engineering limitations, using MBCs poses aesthetic challenges, such as surface color uniformity, making it difficult to achieve a consistent appearance in furniture. The texture of mycelium can also vary, making it difficult to control the final appearance of furniture [11]. A diverse array of appearances are available with bio-based materials, ranging from traditional and rustic options to more contemporary and modern designs [15]. Considering the potential bias against fungi and the specific characteristics of MBCs, the question about this material’s acceptance level among designers and future customers is fully justified. Unfortunately, this issue is not fully addressed in the scientific literature. The key here is the concept of “likeability”, i.e., the answer to whether the consumer will like the material. The “likeability” feature of the material is associated with sensory marketing issues [16]. Even materials with good physical and economic properties may not enter wide industrial applications if users do not accept them [17].

In the case of implementing MBCs-class materials, the risk of non-acceptance of the product is exceptionally high. This material is “growing” and, therefore, is difficult to manufacture—its coloring and surface texture are not regular and homogeneous. Items made of MBCs have a unique aesthetic. Another challenge in implementing MBC materials is their biological origin; the substrate is biological, and the mycelium that holds it together is also biological. The fungus may be of particular concern, despite the use of safe, non-mycotoxic fungi species and their thermal deactivation at the final stage of manufacturing an item from MBCs [13]. These factors narrow down the application field, especially when new materials are introduced for new uses. The purpose of the present research is to answer the following questions:

Are people ready to accept MBCs for direct, everyday use? 

Are they ready to accept MBCs in furnishings or other interior design elements?

Thus, an experimental study of the acceptance level of the mycelium-based composites among designers and future everyday users and the “likeability” of those innovative materials becomes crucial.

## 2. Materials and Methods

### 2.1. Organization of the Research

Each engineering material has a specific set of properties that affect the experience of the person who comes into direct contact with it. Although the human sensory experience uses all senses simultaneously, visual perception takes precedence. Therefore, the visual perception of a new material is usually supraliminal to other senses, owing to which the material is assessed and classified based on its appearance. Nevertheless, in research, the sensitivity of the other senses cannot be overlooked [18]. Although sight provides first impressions, the other senses detail the overall experience and are used in long-term contact with the material. The combined action of several senses gives information complete enough to evaluate the material reliably. Therefore, the initial examination of the material was to determine organoleptic comfort, considering sight, smell, and touch.

Considering the argument presented, three consumer surveys were made to obtain the broadest possible range of information on the studied material. The results of the consumer surveys were correlated with each other to produce generalizations and conclusions. The order of performing the studies and presenting the results were related to the complexity of the subject matter, from the fundamental issues of sensory perception, through personal and professional decisions when choosing between two products.

- Test A: assessing the organoleptic comfort (sight, touch, and smell) of MBCs with a three-degree scale;
- Test B: assessing the MBC product acceptance with a nine-degree scale—determining personal decision (methodology based on [19]);
- Test C: comparing the MBC wall cladding panels with reference panels made of chamotte clay (pairwise comparisons) (methodology based on [20]).

### 2.2. Production of Samples

**Figure 1:**
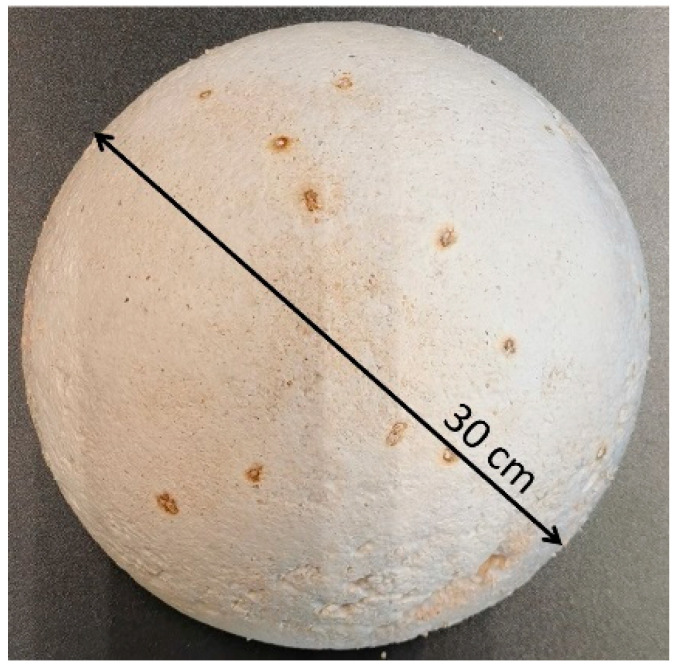
The sample used in test A (photo by A.B.).

It is worth noting that there is no single method of MBCs production (the 2022 review includes an extensive comparative analysis of the applied production conditions based on 92 research articles [13]). Figure 1 presents the samples used for consumer testing in studies A and B. They were made of MBCs and had a hemispherical (dome) shape with a diameter of 30 cm, which allowed the surface to be observed from different angles. The chosen shape highlighted well the texture of the material and changes in Chiaroscuro through varying shell gradients and defined edges.

The first stage in producing all MBC samples was to prepare gypsum molds that would allow multiple pieces of the same shape to be obtained. Afterwards, the molds were thoroughly cleaned and isolated with a polyethylene film. The next step was adding a substrate into the molds and fungus inoculum. Once the molds were filled with the mycelium-infected substrate, they were sealed with another sheet of a polyethylene film. This film was punctured to allow for airflow to the growing fungus. The humidity levels and mycelium growth were closely monitored daily.

**Figure 2:**
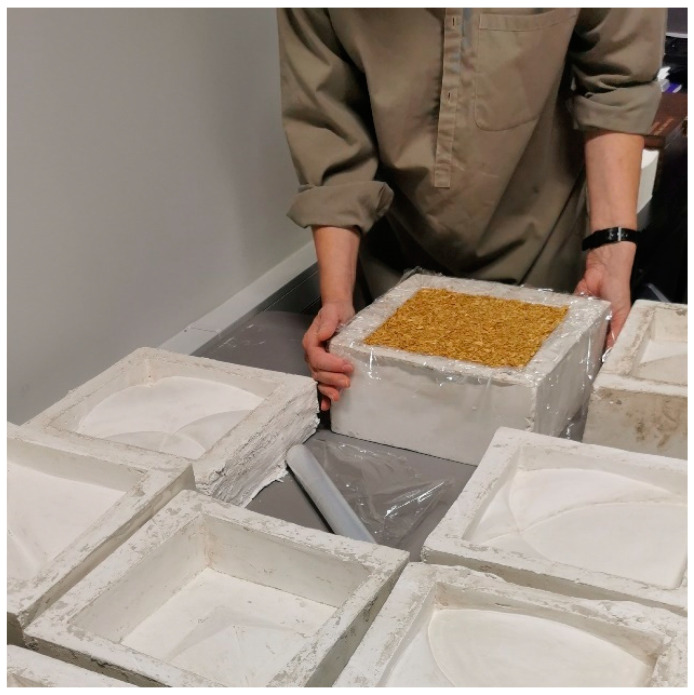
Wall cladding element before ripening—the form filled with MBCs (photo by A.B.).

**Figure 3:**
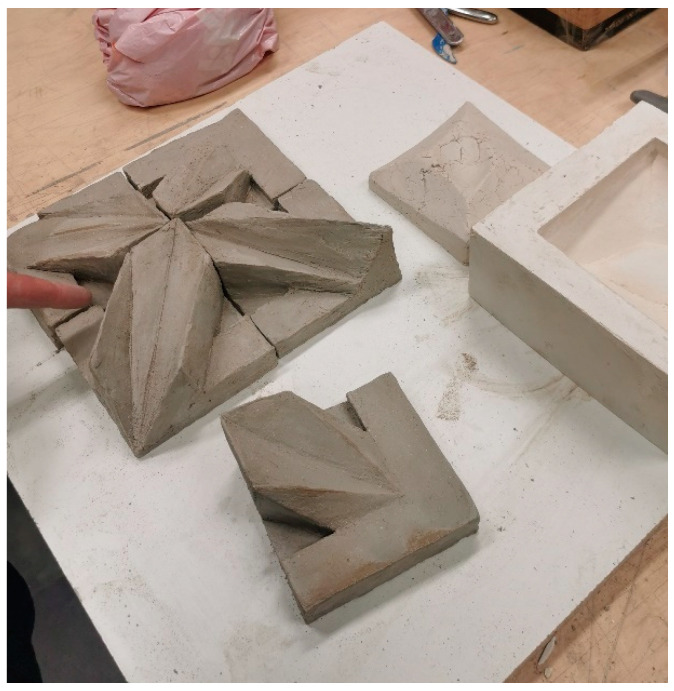
Wall cladding panels made of chamotte clay in realization (photo by K. G.-K.).

On the fourth day of growth, the top film was removed to allow for primary drying, and the fungus maturation continued. On the fifth day, the mycelium grew to fill the mold, and drying was conducted to inactivate the fungus. The molds filled with the substrate to produce MBC samples used in test C are shown in Figure 2. As mentioned, in test C, fired unglazed chamotte clay cladding panels were used as reference samples. These reference samples before firing are shown in Figure 3. 

**Figure 4:**
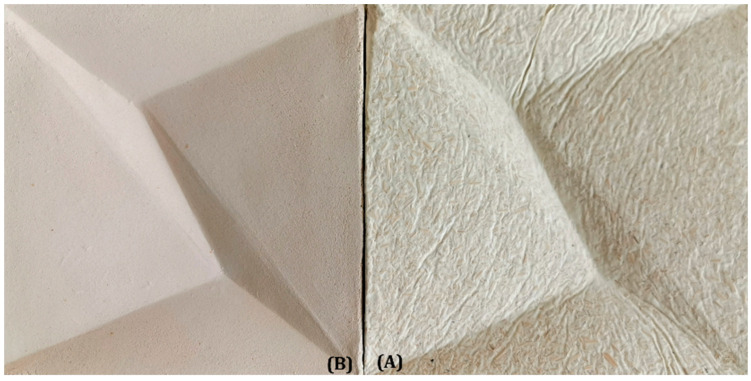
Elements of the wall cladding (size: 20 cm × 20 cm): (**A**) wall cladding made of MBCs; (**B**) wall cladding made of chamotte clay (photo by A.B.).

Figure 4 shows a comparison of the surface structures of both the described materials.

Test C evaluated a specific modular product in a contemporary interior design style. These modules can be put together in any way desired (students designed the studied cladding under the supervision of Klaudia Grygorowicz-Kosakowska, as part of the sculpture class in the second year of the architecture). The clay cladding panels can be used as a bed headboard, a fireplace backdrop, or decorative panels. Chamotte clay products are characterized by their distinct and appealing aesthetics, featuring a natural and heavily grained texture, as well as a soft, beige color. Thanks to its bright and porous surface, chamotte clay did not create contrast with the MBC during an examination. This made it a good material for use alongside MBCs in a similar stylistic context.

### 2.3. Respondents

The survey involved 80 respondents, including 52 females and 28 males, aged 19–24 years, who are the students of the Poznań University of Technology Faculty of Architecture. It is the group of people who will enter the job market as architects and interior designers in the coming years and shape the design trends, impacting the product market. This group’s opinions are considered vital in developing designs that will soon be implemented and enter the market. One of the characteristic features of Generation Z—the Post Millennials to which group the respondents belong—is their sensitivity to sustainability and environmental issues. We respected anonymity while collecting, analyzing and reporting survey data. No personal data were collected, so the data about compared engineering materials were not connected with personal information. Respondents could not influence each other’s responses. All respondents agreed to participate in the study. 

### 2.4. Tests Environment

**Figure 5:**
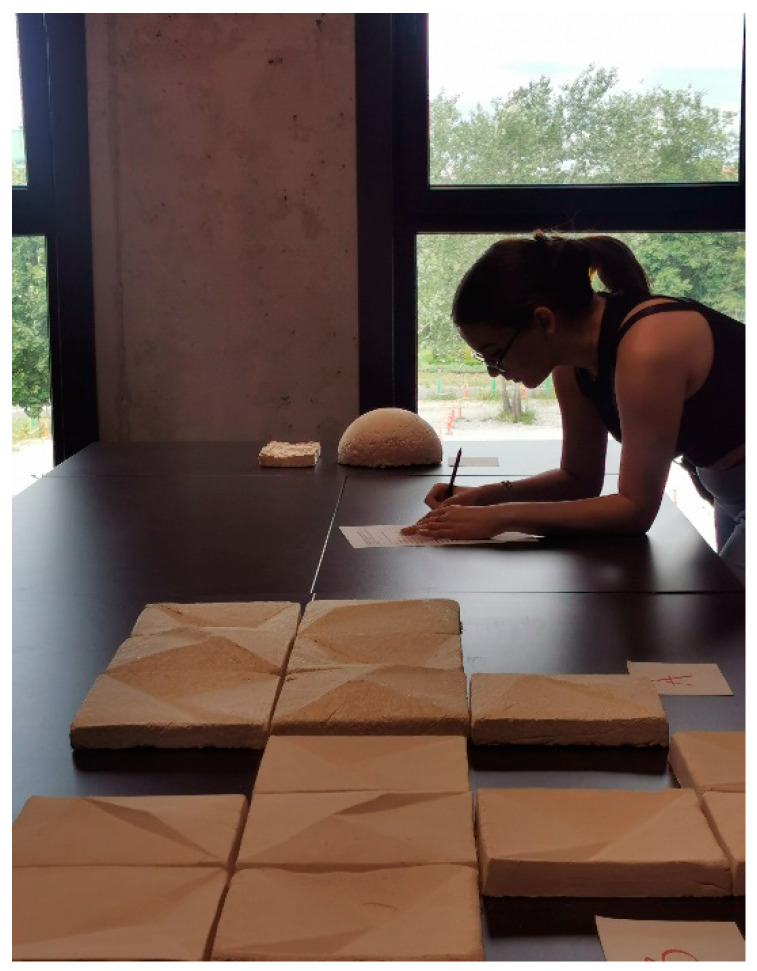
Sample assessment process. Respondents assessed the products individually by typing their answers to the questions on the test forms (photo by A.B.).

**Figure 6:**
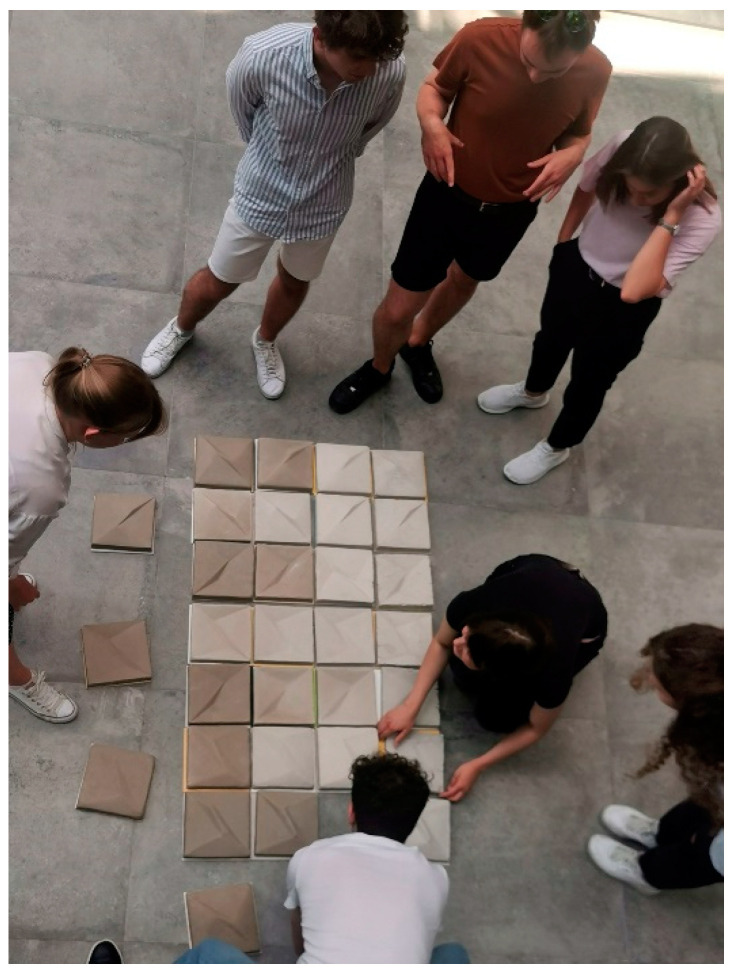
Arranging composition with the wall cladding panels formed in chamotte clay (photo by A.B.).

**Figure 7:**
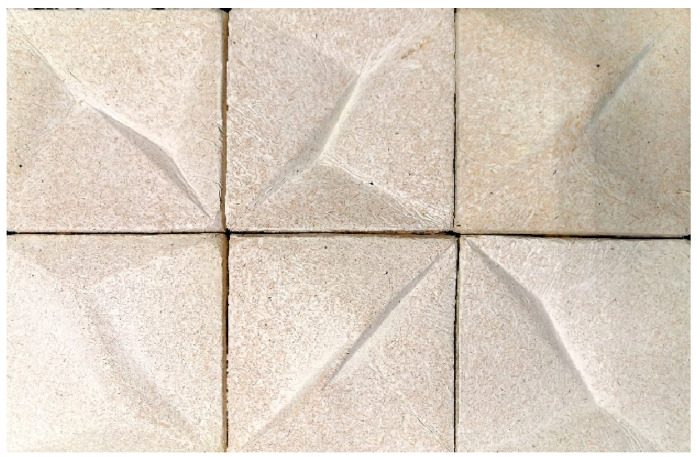
Wall cladding panels made of MBC (photo by A.B.).

The tests were carried out in a room with three individual sample presentation stands, allowing independent evaluation, free from the influence or suggestion of others. Each respondent could access only one stand at a time. The room was thoroughly ventilated before the test. It had a temperature of 22 ± 2 °C and a relative humidity of 60% ± 5%. The samples were assessed at a color temperature of 5000 K to 10,000 K against a neutral, uniform background identical for all the elements presented. The stand for sample evaluation is shown in Figure 5. Figure 6 and Figure 7 show the layout of the wall panels assessed by the respondents.

### 2.5. Experimental

#### 2.5.1. Test A: Consumer Test with a Three-Degree Scale

The first test of three consumer tests (test A) was conducted as an organoleptic assessment, using three senses simultaneously. The assessment involved the properties of the test material perceived in the following manner: visual—in terms of color (pleasant, neutral, and ugly), olfactory (pleasant, neutral, and unpleasant), and haptic (pleasant, neutral, unpleasant/hard, difficult to define/warm, neutral, and cold). The respondents were presented with a hemispherical sample A, which they could touch, see and smell. Five questions were asked about the reception of the material:

1. Do you perceive the material’s color as pleasant, neutral, or ugly?
2. Do you perceive the material as warm, neutral, or cold?
3. Do you perceive the material surface as hard, difficult to define, or soft?
4. Do you find your tactile sensation of it pleasant, neutral, or unpleasant?
5. Do you find your olfactory sensation of it pleasant, neutral, or unpleasant?

The purpose of the questions thus formulated was to determine the level of acceptance at a fundamental, physiological level. 

#### 2.5.2. Test B: Consumer Tests with a Nine-Degree Scale of Material Acceptance

The second test (test B) assessed product acceptance and desirability using a nine-point hedonic scale, typically used in consumer research to measure consumer response to some products [19]. In this test, the respondents again presented the same hemispherical sample and wall panels made of MBCs, which the respondents could touch, see and smell. The respondents were asked questions:

1. Would you accept the material in interior design elements in your own home?
2. Would you accept the material in interior design elements in a home that you design with an ecological aesthetic?

The first question concerned a personal opinion on the material, with positive answers indicating a significant positive reception. The second question, on the other hand, concerned the respondent’s general opinion regarding the use of the material in interiors.

#### 2.5.3. Test C: Consumer Tests with the Method of Pairwise Artifact Comparison of Wall Cladding Samples

The third test used the differential, pairwise comparison method to compare the two products, a piece of wall cladding made of chamotte clay, fired and unglazed, and a mycelium-based composite (MBC). The assessment was carried out to test the potential competitiveness of the solution on the market and to determine whether the new material would gain consumer acceptance and whether it was “likable” compared to other solutions. The aim was to determine the hedonic quality resulting from evaluations of sensory experience in terms of subjective emotions. The respondents presented cladding made from the two materials for comparisons in pairs of the MBC and chamotte clay, with the following questions asked:

1. Which cladding version gives the impression of being eco-friendly?
2. Which cladding version is more original?
3. Which cladding version is more visually appealing?
4. Which version of the cladding is easier to fit into the aesthetics of an ecologically styled interior?
5. Which cladding version is easier to fit into the aesthetics of a modern interior?
6. Which version of wall cladding is more attractive?
7. Which version of wall cladding do you prefer?

The questions, in this case, concerned both the selection of one of the two solutions in terms of the degree of originality, visual appeal, interest, preference, and their potential use, i.e., use for the interior design of a particular style.

## 3. Results and Discussion

### 3.1. Results and Interpretation of Test A: Consumer Test with a Three-Degree Scale

**Figure 8:**
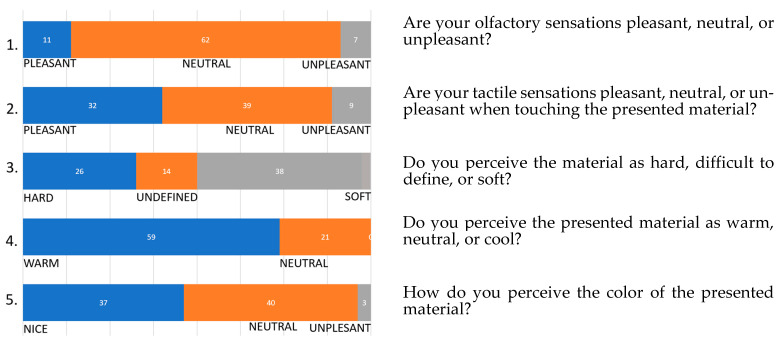
Results of test A—consumer test on a three-degree scale, presented in a cumulative percentage graph.

The results of test A pointed out the generally positive assessment of the MBC material, as the respondents perceived it as neutral or pleasant in all the fields evaluated. The mycelium-based composite’s (MBC) visual quality was not disturbing: 62 out of 80 respondents perceived the color as neutral, 11 perceived it as pleasant, and seven perceived it as unpleasant. When touched, most respondents thought the material was neutral (39 people) or “warm” to the touch. Thirty-six people thought that the material felt soft; the opposite opinion was shared by only 26 people, whereas for 14 respondents, it was difficult to determine its texture. The overall sensation was that the material felt pleasant or neutral when touched. Olfactory sensations were also positive: negatively judged by three people only, against 73 respondents who thought the smell of the MBC was pleasant or neutral (Figure 8). It needs to be stressed that the test was performed in a well-ventilated room which can influence the results in olfactory sensations. 

Graph 1 shows the results of test A—consumer test on a three-degree scale, presented in a cumulative percentage graph. 

The results of test A yielded correlations between the answers to questions 2, 4, and 3. Out of a group of 32 respondents who rated the tactile sensation of the material as pleasant, 26 respondents described it as warm, while 6 respondents described it as neutral. This suggests a correlation between factors 4 and 2 in terms of the tactile properties of the material. No one in this group described the material as cold. Therefore, the material’s advantage is the sensation of “warmth” to the touch. This is vital, when it comes to the expectations of the material. 

However, observing the correlation between questions 2 and 4, 32 respondents rated tactile sensations as positive, 12 respondents declared that they simultaneously perceived the material surface as soft, 12 respondents perceived it as neutral, and 6 respondents perceived it as hard. Therefore, the asset of the material is its “softness”. The correlations between responses to questions 4 and 2 and between questions 4 and 3 indicated that the respondents valued “warm” and “soft” materials. 

Other studies confirm that surfaces that users often like can be described as soft and warm, as well as smooth and warm, indicating the feeling of warmth as a decisive positive characteristic [21]. The described distribution of responses also reminds us of the importance of tactile sensations and their often-underestimated role in the design of functional objects and interiors. Visual perception dominates the evaluation of functional objects and designs, although sight does not determine the complete experience of the material. Research focusing on touch and smell demonstrates that when perceiving with one sense, the user mentally constructs an “image” relevant to the other senses. The drive for a multi-modal, multisensory experience prevails [22].

### 3.2. Results and Interpretation of Test B: Consumer Tests with a Nine-Degree Scale of Material Acceptance

**Figure 9:**
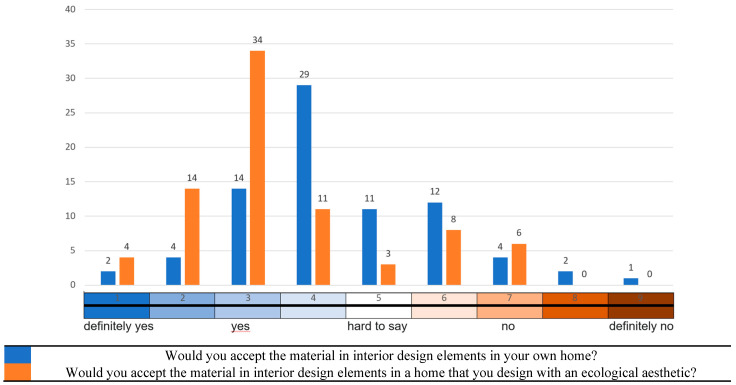
Results of test B—consumer tests on a nine-degree scale of material acceptance.

Question 1, worded in this way, required expressing one’s personal opinion about the material, with positive answers indicating a significant positive reception. The second question, on the other hand, referred to the respondent’s general opinion regarding using the material. The results of test B demonstrated that the respondents’ acceptance of the MBC was high, especially with homes designed by the respondents in ecological aesthetics. The result of each respondent was a preference profile from rank 1 = “definitely yes” to rank 9 = “definitely no”. As shown in Figure 9, the respondents significantly associated the MBC with ecological solutions. However, it is interesting that the respondents did not necessarily associate their homes with environmentalism. Staying eco-friendly is important, but it seems not at one’s own home.

The results shown in Figure 9 suggested that the material presented to the respondents was perceived as environmentally friendly due to its natural characteristic, which was perceivable upon contact with the MBC. Although the respondents did not know the production process, they guessed right that they were dealing with an ecological material. 

The results of research in related areas [23] confirm the above observations. They show that most young people believe that sustainability is the right course of action but that their positive responses are not noticeably correlated with the degree of familiarity with sustainability. Students strongly associate sustainability concepts with their environmental aspects rather than their economic and social aspects. Regarding their participation in “sustainable” lifestyles, they most often mention “slightly green” activities relating to consumer responsibility, such as changing shopping habits, recycling, and saving energy or water. Young people are not optimistic about the future of society in the face of environmental threats.

### 3.3. Results and Interpretation of Test C—Consumer Tests Following the Method of Pairwise Artifact Comparison of Wall Cladding Samples

**Figure 10:**
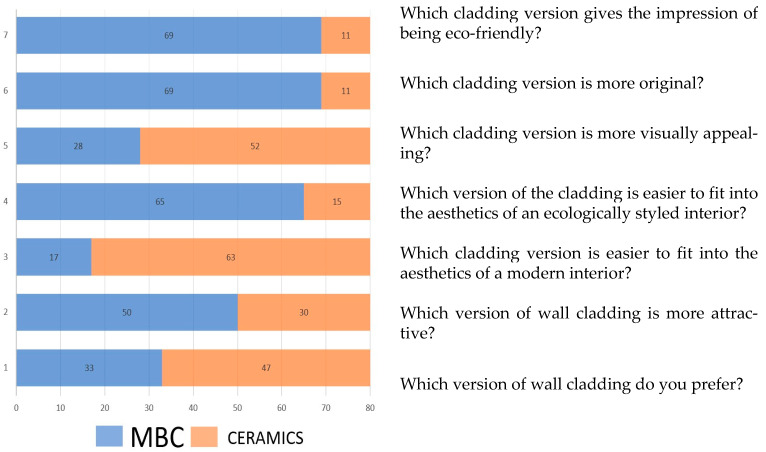
Results of test C—comparison of two decorative claddings: MBC; ceramic casting.

This study expressed preferences for pairwise comparisons, leading to ranking the cladding type by answering seven questions. General responses when following the method of pairwise artifact comparison of wall cladding samples revealed the advantage of ceramic material, mainly regarding the overall assessment (question 1 in test C) and visual appeal (question 5 in test C). The respondents also believed that the chamotte clay cladding would be easier to fit into the aesthetics of a modern interior, which would be better with its style (question 3 in test C). The results of the research are presented in Figure 10. At the same time, mycelium-based composites (MBCs) were clearly perceived as a more attractive and original solution (questions 2 and 6 in test C). It also gives the impression that being eco-friendly and definitely would fit and enhance an ecologically styled interior (questions 4 and 7 in study C). 

An additional discussion is required to juxtapose whether the material is interesting and original with the answers concerning aesthetics, i.e., its visual appeal. Although the MBC was found to be less visually appealing, the respondents found it more interesting, original, and environmentally friendly. Therefore, the pattern of responses in test C indicated a potential paradigm shift in aesthetics to this material.

Interior design and interior decoration mirror a trend known from the clothing industry, i.e., fast fashion. Retail companies specialized in interior design and decoration vigorously promote the vision of often changing collections once or even several times during one season [24]. Fast fashion’s rapid pace has drawn criticism for its detrimental impact on the environment and its role in fueling overconsumption and waste [25]. Consumers are used to permanent purchases, and even the awareness of the problem and negative publicity of fast fashion practices do not always prevail [26]. In this situation, the pro-environmental strategy of using environmentally friendly materials could be implemented along with the policy of reducing consumption, which is highly reasonable.

Several main paradigms or philosophies influence the aesthetics of space in interior design. Some of the most prominent are as follows:

- Minimalism: This philosophy emphasizes simplicity and functionality, focusing on clean lines, neutral colors, and a lack of clutter. A lack of ornamentation and a focus on form and function characterize minimalist interiors.
- Modernism: Modernist interiors are characterized by a focus on functionality, technology, and the use of new materials and construction methods. They often feature simple, clean lines, and a neutral color palette.
- Scandinavian: Scandinavian interior design is known for its simplicity, functionality, and focus on natural materials. This style often features light colors, natural wood, and an emphasis on creating a cozy and comfortable living space.
- Art Deco: Art Deco interiors are characterized by their use of bold geometric shapes, strong colors, and luxurious materials. This style often features metallic accents, such as brass or chrome, and incorporates exotic motifs inspired by ancient cultures.
- Traditional: Traditional interiors are characterized by classic forms, such as ornate moldings, classic furniture styles, and rich colors and fabrics. This style often incorporates antiques and heirloom pieces and is designed to evoke a sense of timeless elegance.

Each of these paradigms has its own distinct aesthetic, and interior designers may use elements from several different paradigms in order to create a unique and personalized design for a space. There are several trends in contemporary aesthetic discourse in the design of functional objects. The leading trend still comes from the modernist tradition of the early twentieth century and is based on the imperative to use the latest achievable materials in streamlined, reduced, and functional forms [27]. The 1970s is the time when the high-tech trend associated with an emphasis on the primacy of technology evolved. These trends have cemented the vision of the design of the future as technologically advanced design forms with perfectly smooth surfaces and precise edges [28]. This classical vision, firmly embedded in the broad public consciousness, has been reinforced by the proliferation of digital (parametric) tools in design, prototyping, and production [29].

At the same time, however, nature-inspired concepts developed in the design arts from the 1930s onwards, including biomorphism, which became the basis for the later ideas of bio-aesthetics and bio-design. The widespread dissemination of knowledge on climate change was initiated by international climate conferences and dates back to 1979 [30]. However, the resulting designs for functional objects were rarely seen and defined as “designs of the future”. The same is true today—despite the attention that eco-design is receiving [31,32], biomorphic forms are gaining popularity in new marketing ideas [33,34] and the demand for sustainably produced product components is not diminishing [35]. Environmentalism is still not seen as a solution of the future. In practice, bio-aesthetic design involves using natural materials, such as wood, annual plants, and stone, as well as incorporating plants and other elements of nature into the interior space. It also involves lighting and color in ways that support circadian rhythms and promotes positive moods. Bio-aesthetics aims to create spaces that are not only aesthetically pleasing, but also are supportive of human health and well-being. 

Within the domain of ecological design itself, the use of animate systems is undoubtedly an interesting concept. Bioengineering handles the creation and construction of “animate” materials at the micro-scale. An example of such a material on a macro-scale are the objects made of MBCs presented herein. There are other, less popular, yet interesting ways of “animate” production, i.e., the solutions of the Full Grown Furniture company, which has found that excellent results can be achieved by growing trees directly in special molds. Full Grown Furniture’s chairs are already design icons, but the relatively small scale of their production makes them luxurious [36].

As presented in the above study, the shift from the imperative of the perfect finish to naturalness is an important signal, suggesting a paradigm shift in aesthetics towards more sustainable solutions. Perhaps, instead of associating modernity with perfectly finished surfaces, designs with more unique surfaces will be accepted, e.g., resulting from the natural growth of the material. In this context, of course, interdisciplinarity and openness to hybrid forms of creation—greatly extending material and technological possibilities—are highly vital [37].

The gap between the answers to questions 1 and 2 in test B (Figure 9) indicated a double standard. MBCs were accepted in general but not in one’s own home. It followed that MBCs were perceived as clearly ecological, but at the same time, they raised some concerns. There could be several potential reasons for the lack of consumer acceptance for interior design products made of these mycomaterials:

- Conviction about riskiness: the natural origin of these materials and the fear of fungus can be challenging;
- Unproven ecological benefits: consumers may be unaware of MBCs and their benefits, such as their sustainable and eco-friendly nature;
- Perceived high cost: MBCs are still relatively new and are not yet widely available. As a result, consumers may perceive the cost of producing and using MBCs in interior design products as high, which could deter them from purchasing these products;
- Personal aesthetic preferences: the respondents may have specific aesthetic preferences regarding interior design, and MBCs may not fit their style or taste (as mentioned, the natural and organic look of these mycomaterials may not appeal to everyone.);
- Inaccessibility: The availability of interior design products made of MBCs is now limited, which could make it difficult for consumers to find these products in stores or online. This could lead to a lack of awareness and interest in mycomaterials among consumers;
- Material properties are unknown. The respondents may have concerns about the durability and performance of MBCs compared to those of traditional materials. They may worry that mycomaterials will not hold up over time or will not perform as well as other materials in certain conditions.

Overall, it may take time for MBCs to gain wider acceptance among consumers in the interior design space. Increasing awareness and education about the benefits and properties of mycomaterials and making these products more widely available and affordable help increase their popularity and adoption among consumers.

## 4. Summary and Conclusions

There are some aesthetic challenges associated with using mycelium-based engineering materials in furniture design:

- Color uniformity: mycelium is a natural material whose color can vary, making it difficult to achieve a consistent appearance in furniture;
- Surface texture: the texture of mycelium can also vary, making it difficult to control the final appearance of furniture.

Studying the material properties of manufactured products in the context of introducing new materials and applying the knowledge to industrial design is a challenge in product design. Due to the subjectivity and different nature of the user’s needs, it is not easy to accurately assess and quantify these characteristics. In this article, the authors relied on usability testing and used traditional marketing and decision-making theory methods, i.e., pairwise comparison and one-step consumer tests on a three- and nine-point scales. As a result, this approach has provided data that helped to understand and identify the requirements of future users.

1. The overall positive evaluation of the mycelium-based composite (MBC) among architecture and interior design students aged 19–24 years, i.e., Generation Z, demonstrated that continued research into the material in question could yield good commercialization results in the coming years. The main observation is that the younger generation of designers showed a high level of acceptance for the material itself and its products. According to study participants, the MBC material can be described as “likable” (test A) and highly ecological (test B). The wall cladding made of the MBC had advantages regarding its uniqueness, its consistency with eco-styled interiors, and the fact that it is interesting (test C). Further considerations should be given to optimizing the properties and its new applications that are not obvious today.
2. The results of the experiments suggest double standards in the respondents. MBCs were generally accepted, but not in their own homes. It followed that MBCs were perceived as clearly ecological, but at the same time they raised some concerns. The fear of fungus is deeply ingrained in many cultures and can lead to skepticism or aversion towards products made from mycelium. Some people may be hesitant to use MBCs in their homes or in products they consume due to concerns about fungal growth and associated health risks. Additionally, MBCs are a relatively new technology, and there is still much to learn about their properties, durability, and potential applications. This can lead to uncertainty and skepticism among consumers and industry professionals alike.
3. Working with this material and other bio-materials can lead to a paradigm shift in aesthetics in which the design mainstream has hitherto been defined by high technology and highly sophisticated design and production methods, which will perhaps soon take on a more casual, nature-like form.

Human acceptance of mycelium-based engineering materials as furniture materials is still growing. However, some people are unfamiliar with mycelium-based materials and may be hesitant to use them in their homes or workplaces. However, there is a growing interest in sustainable and eco-friendly materials, and mycelium-based materials have the potential to appeal to this market. Additionally, as people become more educated about mycelium-based materials’ environmental benefits and unique aesthetic qualities, they may be more likely to accept and embrace them.

It’s also worth noting that acceptance can vary by cultural and regional factors. In some regions, there may be a greater appetite for experimental and unconventional materials, while in others, there may be more traditional preferences. To sum up, human acceptance of mycelium-based engineering materials will likely continue to grow as more people become familiar with the material and its benefits, but individual preferences and cultural factors will also influence it.

## Data Availability

All data obtained during the research are included in the article.
